# Supplementary material for: MetaRibo-Seq measures translation in microbiomes
Source: Nat Commun. 2020 Jun 29;11:3268. doi: 10.1038/s41467-020-17081-z (PMC7324362; doi:10.1038/s41467-020-17081-z)
Supplement: Supplementary file 10 — Supplementary Data 7 [file 41467_2020_17081_MOESM10_ESM.zip › File2/Confidence_High_Taxonomy/265197_out.krona.html]

Javascript must be enabled to view this page.

members
magnitude
magnitudeUnassigned
count
unassigned
taxon
rank

265197\_out

7

7
2
superkingdom

1239
phylum
7

7
186801
class

7
order
186802

186806
family
6

6
1730
genus

165185
species

SRS019068\_contig\_number\_49809SRS049712\_contig\_number\_contig-100\_7062.37345SRS053214\_contig\_number\_contig-100\_12468.54795SRS143342\_contig\_number\_contig-100\_28779.70291SRS144183\_contig\_number\_11740SRS148159\_contig\_number\_34432
6

1
186803
family

2569097
genus
1

1

SRS148721\_contig\_number\_contig-100\_6336.318972
species
39488
